# Antimicrobial Efficacy and Soft-Tissue Safety of Air-Polishing Powders in Periodontal Therapy: A Narrative Review

**DOI:** 10.3390/jfb17010009

**Published:** 2025-12-22

**Authors:** Ștefania Sorina Ifrim, Alina Bârdea, Alexandra Roman, Andrada Soancă, Silviu Albu, Anda Gâta, Carmen Silvia Caloian, Andreea Cândea

**Affiliations:** 1 Department of Periodontology, Applicative Periodontal Regeneration and Pediatrics Dental Medicine Research Center, Iuliu Hatieganu University of Medicine and Pharmacy Cluj-Napoca, Victor Babeș St., No. 15, 400012 Cluj-Napoca, Romania; stefania.sori.hangan@elearn.umfcluj.ro (Ș.S.I.); alina.stanomir@umfcluj.ro (A.B.); andrada.popovici@umfcluj.ro (A.S.); caloian.carmen.silvia@elearn.umfcluj (C.S.C.); andreea.ciurea@umfcluj.ro (A.C.); 2 Emergency County Clinical Hospital, Clinicilor St., No. 3-5, 400347 Cluj-Napoca, Romania; 3 2nd Department of Otolaryngology, Iuliu Hatieganu University of Medicine and Pharmacy Cluj-Napoca, Republicii St., No. 18-20, 400015 Cluj-Napoca, Romania; silviualbu63@elearn.umfcluj.ro (S.A.); gataanda@elearn.umfcluj.ro (A.G.)

**Keywords:** air-polishing, periodontitis, biofilm removal, glycine, erythritol, cells, antimicrobial therapy

## Abstract

Periodontitis is a biofilm-induced multifactorial disease characterized by non-reversible damage of the periodontal tissues. Dysbiosis of the subgingival microbiota plays a crucial role in periodontitis. In this regard, conventional periodontal treatment consists of subgingival mechanical instrumentation, but adjunctive methods, such as air-polishing powders, have also sparked considerable interest due to their ability to efficiently disrupt biofilm with minimal tissue damage. The aim of this narrative review is to provide an overview and critical discussion of the recent literature on the properties and interactions of air-polishing powders with oral bacteria and soft tissues. Fifteen studies were included. Eight recent clinical studies demonstrate that air-polishing powders (e.g., glycine, erythritol) can significantly reduce periodontal pathogens, thereby supporting their role in effective biofilm control; In vitro evidence from four included studies indicates cell-type-specific responses to different powders, with trehalose demonstrating superior biocompatibility compared with glycine and erythritol/chlorhexidine formulations. This variability highlights the importance of choosing the right powder to improve clinical outcomes and reduce tissue side effects. By integrating microbiological, cellular and histological findings, the objective of this review is to clarify the antibacterial efficacy and biocompatibility of air-polishing powders. Overall, air-polishing powders have been shown to be safe and effective as an adjunctive treatment, in both active periodontal and supportive periodontal therapy.

## 1. Introduction

Periodontitis is a highly prevalent chronic disease affecting a significant proportion of the general population, primarily triggered by a dysbiotic shift in the subgingival microbiota and subsequent persistent periodontal inflammation [1,2,3,4,5]. Subgingival mechanical instrumentation (scaling and root planning, SRP) still represents the gold standard of periodontitis treatment, demonstrating efficacy in improving clinical outcomes across most cases [6]. However, conventional SRP therapy has limited efficacy in certain circumstances and unclear long-term effects [5] related to tooth anatomy, subgingival biofilm virulence and host immune particularities [7]. Efforts to optimize treatment efficacy include several adjunctive approaches, which have subsequently shown limited effectiveness in clinical settings [8,9,10,11]. Moreover, the escalating rise in antibiotic-resistant oral pathogens and the broader push to minimize unnecessary antimicrobial use [12] favor the exploration of alternative or adjunctive strategies for managing periodontal biofilms [13].

Guided biofilm removal has emerged as an interesting concept and technique in periodontitis treatment, removing the biofilm by visualizing it with disclosing agents and subsequently eliminating it with specialized air abrasive powders [14]. Glycine and erythritol powder air-polishing approaches are designed as minimally invasive techniques to enhance subgingival biofilm removal in daily practice [14]. Trehalose and bioactive glasses have also been promoted more recently as air-polishing agents [15,16]. In parallel, sodium bicarbonate air-polishing can be used to manage supragingival microbial accumulations [14]. The potential antibacterial activity of different air-polishing powders on residual bacteria may offer an added therapeutic benefit to mechanical elimination [17], although some inconsistencies on this property are reported by the literature. Glycine-based air-polishing significantly reduced subgingival bacterial counts as compared with hand instrumentation [18], with a 30% reduction in surviving biofilm cells after the treatment [19]. Erythritol air-polishing powder has gained clinical acceptance, demonstrating positive outcomes in periodontal treatment comparable to traditional methods [20]. Studies suggest that erythritol powder may have a prolonged antimicrobial effect on subgingival biofilm, potentially reducing the amount of some periodontal pathogens during periodontal treatment [21]. On the other hand, others reported no significant differences in microbiological outcomes between subgingival debridement methods [22,23].

While air-polishing powders are appealing for their ease of use and high patient acceptance, uncertainties remain regarding their effects on subgingival biofilms [18,19,22] and their overall clinical efficacy [23]. These concerns are compounded by their absence from the current Stage I–III periodontitis clinical guidelines [6] and by reports of potential traumatic effects on soft tissues [24]. Taken together, these factors raise questions about the strength of evidence supporting their routine use in clinical practice.

This narrative review aims to systematically synthesize and critically evaluate recent evidence regarding the properties and interactions of air-polishing powders with oral bacteria and soft tissues, including aspects such as antibacterial action, biocompatibility, and related adverse effects. The goal is to support the clinical integration of these powders as adjunctive tools in periodontal care, based on robust scientific evidence. Additionally, the review highlights current research limitations and proposes future directions to advance knowledge in this field, as while recent studies highlight the clinical benefits of air-polishing, its use in everyday periodontal practice remains variable and generally limited to clinicians familiar with minimally invasive biofilm-control approaches, which may explain why it has not yet been incorporated into the current Stage I–III periodontitis treatment guidelines.

## 2. Methods

Publications from January 2020 to August 2025 were considered for this narrative review. Searches were conducted in PubMed, Cochrane Library and Scopus, using a combination of MeSH terms and related keywords linked by Boolean operators (**AND**, **OR**) to combine concepts and synonyms. The main terms included “*Periodontitis*”, “*Periodontal Treatment*”, “*Air-Polishing*”, “*Air-Polishing Powder*”, “*Glycine*”, “*Erythritol*”, “*Trehalose*”, “*Sodium Bicarbonate*”, “*Oral Bacteria*”, “*Porphyromonas gingivalis*”, “*Oral Tissue*”, “*Oral Cells*”, “*Fibroblast*”, “*Epithelial Cell*”, “*Biocompatibility*”, “*Histology*”, and “*Tissue Abrasions*”. Quotation marks were used for exact phrases, and the asterisk (*) served as a truncation symbol to capture word variations.

Eligible studies included randomized clinical trials (RCTs), controlled clinical trials (CCTs), cohort studies, in vitro or animal studies, and systematic reviews with or without meta-analyses. Titles and abstracts were screened independently by four reviewers (Ș.S.I., A.R., C.S.C., and A.C.), and full texts were retrieved when all reviewers agreed on eligibility. Additionally, the reference lists of included studies were examined to identify further relevant articles. Eight clinical studies, one ex vivo study, four in vitro studies and two animal model studies were included. Searches were limited to articles published in English.

Exclusion criteria: articles published prior to 2020, studies reporting outcomes outside the aim of this review, those with insufficient or missing data, monographs or letters to the editor, and publications in languages other than English.

The findings of this review are presented in thematic sections to provide an updated and comprehensive overview of the topic.

## 3. Overview of Air-Polishing Powders

### 3.1. Characteristics and Properties

The fundamental working principle for air-polishing devices involves delivering a pressurized slurry composed of abrasive powder particles mixed with water through a specialized nozzle system [25].

These systems are equipped with one of two types of nozzles, each suited to specific clinical applications, more specifically, to work on supragingival and on subgingival zones. The standard supragingival nozzle is intended for removing plaque and extrinsic stains from above the free gingival margin. Early air-polishing subgingival devices were equipped with a nozzle featuring two concentric channels, with the air–powder mixture exiting through the inner lumen and water delivered through the outer lumen [25]. In contrast, a newly designed subgingival nozzle enables effective debridement of periodontal pockets deeper than 5 mm [14]. It features two lateral outlets positioned about 2 mm coronally from the tip, directing the air–powder jet horizontally toward the root surface and pocket epithelium. A third outlet, located at the tip, releases only a water spray, providing irrigation and helping remove loosened biofilm and debris. [26].

Air-polishing systems are used with various oral powders, such as sodium bicarbonate, glycine, erythritol, or trehalose, which can be classified according to particle size, shape, chemical composition, and abrasiveness, factors that determine their suitability for supragingival or subgingival applications [26].

Sodium bicarbonate (NaHCO_3_) is a non-toxic, water-soluble powder that is highly effective for the removal of supragingival plaque and heavy extrinsic stains [27]. Sodium bicarbonate has been reported to dissolve mucus and loosen debris accumulated around the teeth when used as a cleaning agent [28], and it also raises the oral pH, preventing overgrowth of aciduric bacteria [29]. In the meantime, sodium bicarbonate reduces colonization by yeast [29]. Although sodium bicarbonate powder is effective for removing plaque and stains, extended use may lead to enamel abrasion and increased surface roughness on restorative materials [16].

Glycine is a low-abrasive amino acid consisting of non-toxic, biocompatible organic salt crystals that slowly dissolve in water [27]. It is approximately 80% less abrasive than sodium bicarbonate [14], and is considered both safe and effective for biofilm removal from tooth surfaces and restorative materials, indicating it for both supra- and subgingival application [30].

Erythritol, a chemically neutral, non-toxic, water-soluble polyol widely used as an artificial sweetener, has a particle size similar to or smaller than glycine, enhancing its stability and tissue compatibility [14]. The commercially available erythritol-based powder has 0.3% chlorhexidine diacetate added at the factory [31]. It is effective in subgingival biofilm removal, and has some antimicrobial effects [32].

Trehalose is a non-cariogenic disaccharide approved for use in food processing [33]. Trehalose is a highly water-soluble molecule with a pH of 6.4; it has a lower abrasive effect on tooth substance than glycine [34].

Tagatose is a ketohexose that provides approximately 92% of the sweetness of sucrose while contributing only 38% of its caloric value [35]. Research has primarily focused on its anticariogenic properties, particularly its ability to inhibit *Streptococcus mutans* biofilm formation and thereby reduce caries risk [36].

Bioactive glasses are biocompatible, non-toxic, non-inflammatory, and non-immunogenic materials capable of interacting directly with living tissues and forming stable chemical bonds. Upon dissolution, they promote the deposition of hydroxyapatite- or fluorapatite-like layers on the tooth surface. Hydroxyapatite may occur naturally, derived from bone tissue [37], or may be synthesized from bioactive glass; in both cases, it is composed mainly of calcium and phosphorus, with small amounts of microelements such as magnesium and sodium [27].

The introduction of these degradable, less abrasive powders, together with advanced subgingival delivery devices, now enables the effective cleaning of deeper periodontal pockets and interdental areas.

A summary of the available powders, together with their key physical properties and clinical indications, are listed in Table 1.

### 3.2. Side Effects

Although air-polishing powders are used in daily practice, there are some limitations to their use. Air-polishing should be used with caution in patients who have difficulty breathing or swallowing. Additionally, it should never be used near surgical wound areas or areas with periodontal pockets with extensive bone loss, as it may risk facial emphysema [26]. In spite the fact that sodium bicarbonate was the first powder to be widely used and is effective for removing supragingival plaque and stains, it is highly abrasive to root cementum, dentin and restorations surfaces [38,39]. Also, different studies contraindicate sodium bicarbonate air-polishing in conditions like hypertension, respiratory disorders, renal insufficiency, Addison’s disease, metabolic alkalosis or with patients taking medication such as potassium, anti-diuretics or corticosteroids [26]. Glycine and erythritol have been developed as less abrasive alternatives, but concerns have been raised by residual powder fragments detected on treated surfaces and in periodontal tissues [40,41]. These residues may alter the surface biocompatibility and also interfere with the healing responses [42]. Also, a study demonstrated that air-polishing with glycine powder resulted in fewer areas of gingival erosion compared to hand instrumentation or air-polishing with sodium bicarbonate [18]. However, air-polishing powders have a good safety profile overall, and only a few pieces of evidence indicate that air-polishing devices may occasionally be associated with uncommon adverse events, such as subcutaneous emphysema and abrasion of the root cementum [23].

## 4. Air-Polishing Powders: Mechanisms of Action in Biofilm Removal

### 4.1. Mechanical Disruption

Air-polishing removes dental biofilm through the propulsion of abrasive particles suspended in a compressed air–water slurry, delivered via a handpiece nozzle. When directed at the target surface, this stream disrupts the organized biofilm matrix by breaking apart the extracellular polymeric substance and physically detaching adherent microorganisms from tooth and root surfaces [26].

The kinetic energy of the air-polishing jet stream is determined by device-specific parameters such as air pressure, water-to-powder ratio, and particle velocity [26]. The ability of air-polishing powders to remove biofilm, calculus, or tooth structure is influenced not only by particle characteristics but also by the angle at which the abrasive is propelled through the pressurized water jet [43]. Higher pressure and water settings generally enhance the cleaning efficiency of air-polishing devices. Water is thought to improve the action of abrasive powders by helping dislodge particles embedded in the surface. Conversely, some authors suggest that the formation of a water film on the target surface may reduce the abrasive effect [27]. It is worth mentioning that water’s kinetic energy breaks the particles and reduces their size, hence adversely affecting the efficiency [43].

Powder properties such as particle size, morphology, and hardness play a major role in determining both abrasiveness and the overall performance of the jet stream [44]. In addition, the quantity of powder loaded in the chamber significantly influences the emission rate, thereby affecting the efficiency and effectiveness of air-polishing procedures [45].

Air-polishing effectiveness is influenced by time duration. Instrumentation time is basically user-dependent, and it may adversely affect the hard or soft tissues [43]. Complete plaque removal was achieved within 5–10 s of glycine air-polishing powder application [18].

Subgingival air-polishing appears to be more effective in removing subgingival plaque than conventional debridement methods, achieving an immediate reduction of approximately 98% in viable bacterial counts following treatment [46].

**Table 1:** Physical properties and clinical indications of air-polishing powders.

Air-Polishing Powders	Composition	Particle Size (µm)	Particle Shape	Solubility	Abrasiveness	Clinical Indications
**Sodium bicarbonate (NaHCO_3_)** **[14,47]**	Sodium bicarbonate (non-toxic)	1–250 (mean particle size 40–65)	Angular, chiseled, sharp edges	Water-soluble	High-abrasive	Supragingival biofilm and early calculus removal
**Glycine** **[14,47]**	Amino acid (non-toxic, organic salt crystals)	25–65 (mean particle size 25)	Less angular, round edges	Slow water-solubility	Low-abrasive (~80% less than NaHCO_3_)	Supra- and subgingival biofilm removal; use on teeth, implants, soft tissue and orthodontic appliance
**Erythritol** **[14,47]**	Sugar alcohol, polyol (non-toxic, chemically neutral)	14–31 (mean particle size 14)	Fine and extra-fine rounded particles	Water-soluble	Minimally abrasive	Supra- and subgingival biofilm and young calculus removal; prophylaxis treatments (gingivitis, periodontitis); primary and secondary supportive periodontal therapy; non-surgical treatment of peri-implantitis and mucositis, implant maintenance; fixed orthodontic appliance; tongue and soft tissue cleaning
**Trehalose** **[34,48]**	Disaccharide (non-toxic)	30 and 65	Fine particles	Water-soluble	Low-abrasive	Supragingival cleaning and subgingival biofilm removal; extrinsic discoloration removal; use in supportive periodontal therapy
**Tagatose** **[35,49,50]**	Non-cariogenic sugar	15	Fine particles	Water-soluble	Low-abrasive	Supra- and subgingival cleaning
**Bioactive glass** **[16,37]**	Glass-based bioactive particles (silica-based, bioactive, non-toxic)	1–10	Regular uniform shape	Low-water-soluble	Low-abrasive	Supra- and subgingival biofilm removal; remineralization of dental tissue

Abbreviations: µm, micron; NaHCO_3_, sodium bicarbonate.

Glycine air-polishing powders have been shown to remove biofilm more effectively than sodium bicarbonate powders, which are inherently more abrasive. When used for subgingival root debridement, glycine powder achieves efficient plaque removal [18]. Also, some studies have reported that glycine may modulate inflammation and immune responses across diverse cell types [51,52], although the mechanisms underlying these actions are not yet fully understood [53]. Growing evidence also suggests that glycine can protect cells from oxidative stress-induced inflammatory responses [54]. Moreover, clinical studies have reported that glycine and erythritol air-polishing powders reduced bleeding on probing and gingival crevicular volume, findings which are consistent with local anti-inflammatory effects [55].

### 4.2. Microbiological Findings

Beyond the physical disruption to the extracellular biofilm matrix, recent studies [17,21,56,57,58,59,60,61,62] have highlighted the microbiological effects of air-polishing powders—particularly glycine and erythritol—in modulating both the total bacterial load and the qualitative composition of subgingival communities (Table 2).

**Figure 1:**
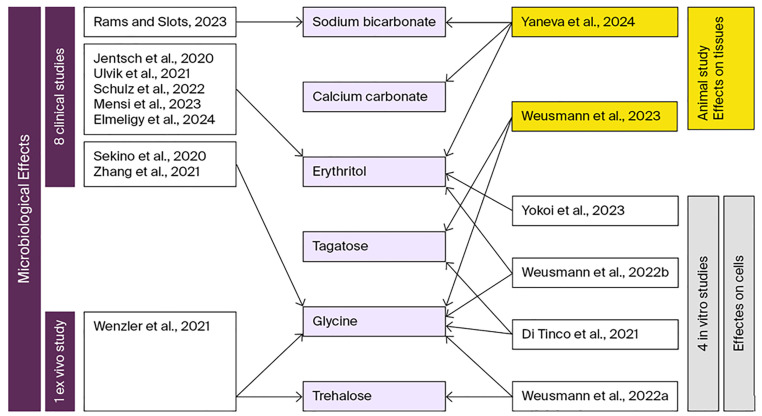
Distribution of studies according to study design and reported microbiological effect, as well as impact on cells or tissues. Light purple shapes denote the types of air-polishing powders evaluated; dark purple shapes represent the labels of clinical and ex vivo studies reporting microbiological outcomes; gray shapes indicate the labels of in vitro studies assessing powder effects on cellular responses; yellow shapes correspond to animal studies assessing the impact of air-polishing powders on soft tissues; white shapes display the references for all studies other than animal studies; black arrows illustrate the association between individual references and the specific air-polishing powder(s) investigated [15,17,21,24,31,35,42,50,56,57,58,59,60,61,62].

We identified seven RCTs, one clinical study, and one ex vivo study published between 2020 and 2025 that used glycine, erythritol, or sodium bicarbonate and included microbiological assessments as a secondary outcome (Figure 1).

One RCT treated 42 periodontitis patients with subgingival sonic instrumentation, either alone or in combination with erythritol air-polishing [56]. *Aggregatibacter actinomycetemcomitans* was rarely detected at any follow-up point. *Porphyromonas gingivalis* counts decreased at the three-month follow-up in the control group. In contrast, *Tannerella forsythia* and *Treponema denticola* counts were significantly reduced after six months in the erythritol group compared with baseline, while no significant changes occurred in the control group. No statistically significant differences were observed between the two groups at any time point [56].

Another study evaluated 19 maintenance patients with recurrent pockets treated either with glycine powder air-polishing or with water-based polishing, each applied two or four times within 90 days [57]. Both groups treated with glycine powder air-polishing showed a decreasing trend in *Porphyromonas gingivalis* up to day 90, with a non-significant rebound by day 180. In all treatment groups, *Tannerella forsythia* tended to decline, and *Treponema denticola* showed a consistent but non-significant reduction over a 180-day study period. No significant intergroup differences were observed [57].

The treatment of 41 stage II to IV periodontitis patients with one approach, such as full-mouth SRP, full-mouth SRP plus immediate glycine air-polishing, or full-mouth SRP after glycine air-polishing, induced a decrease in subgingival counts of *Porphyromonas gingivalis* and *Aggregatibacter actinomycetemcomitans* at 24-h and 3-month points compared to baseline [58]. After 24 h, *Aggregatibacter actinomycetemcomitans* and *Fusobacterium nucleatum* counts were reduced mostly in patients treated with glycine-based approaches. At six-weeks follow-up, *Porphyromonas gingivalis* levels were significantly reduced only by glycine air-polishing applications before SRP [58].

Another trial compared erythritol air-polishing to conventional ultrasonic and manual instrumentation applied for furcation sites in maintenance patients [59]. The targeted detection of “red complex” species (*Porphyromonas gingivalis*, *Treponema denticola*, *Tannerella forsythia*) and other pathogens (*Aggregatibacter actinomycetemcomitans*, *Prevotella intermedia*, *Fusobacterium nucleatum* subspecies, *Parvimonas micra*, *Prevotella nigrescens*) revealed no significant differences between groups at baseline or six or twelve months. Both groups showed an increasing trend for *Treponema denticola* and *Parvimonas micra* from baseline to six months [59].

An ex vivo study investigated the effectiveness of glycine-based and trehalose-based air-polishing powders for bacterial removal in experimental subgingival periodontal pockets of porcine mandibles, using biofilm-coated titanium samples. The lowest residual bacterial counts were observed in the glycine group. Both glycine- and trehalose-based powders significantly reduced the total bacterial load compared with untreated controls. Relative to the initial bacterial load, reductions of 99.0%, 93.3%, and 77.6% were achieved following glycine, trehalose, and water treatment, respectively. All tested approaches reduced the levels of major periodontal pathogens, including *Porphyromonas gingivalis*, *Fusobacterium nucleatum*, *Campylobacter rectus*, *Aggregatibacter actinomycetemcomitans*, and *Prevotella intermedia* [17].

Another RCT evaluated 40 patients with stage III–IV periodontitis treated with one of four approaches: quadrant-wise scaling and root planing (SRP), full-mouth SRP within 24 h, full-mouth SRP plus antiseptic application, and full-mouth SRP plus antiseptic application combined with subgingival erythritol air-polishing [60]. Full-mouth approaches led to reductions in subgingival genera associated with periodontitis, such as *Fusobacterium*, though recolonization occurred by six months. A similar trend was noted for uncultured *Prevotella* species in the erythritol group [60].

In another RCT, a real-time quantitative PCR microarray capable of detecting 93 microbial species evaluated subgingival bacterial changes in 40 patients with stage III–IV periodontitis treated either with subgingival ultrasonic instrumentation alone or with ultrasonic instrumentation combined with erythritol–chlorhexidine air-polishing [61]. At baseline, both groups presented high levels of periodontopathogens alongside other oral species. A significant reduction in *Treponema denticola* was observed after three months in the erythritol–chlorhexidine group. Other species associated with periodontitis—such as *Actinomyces israelii*, *Filifactor alocis*, *Porphyromonas endodontalis*, *Tannerella forsythia*, and *Treponema socranskii*—showed significant decreases, particularly in female patients and non-smokers of the test group. Although non-significant overall, reductions in these species were more pronounced in the test group than in controls after three months [61].

Supragingival sodium bicarbonate air-polishing was assessed for its impact on subgingival microbiota in 15 patients with stage III periodontitis [62]. Immediately after treatment, an 84.9% mean reduction in total subgingival viable counts was reported, along with significant decreases in *Prevotella intermedia*/*nigrescens*, *Fusobacterium nucleatum*, and total red/orange complex species per patient. Subgingival motile morphotypes were reduced by 85.3%, from a pre-treatment mean of 17.7% to 2.6% [62].

In patients with stage I–II periodontitis, SRP alone, SRP plus subgingival erythritol air-polishing, or SRP plus diode laser were compared [21]. The erythritol group showed a significant immediate increase in *Aggregatibacter actinomycetemcomitans* and a decrease in *Porphyromonas gingivalis*, but no notable changes at two, four, or six weeks [21].

Glycine air-polishing performed prior to SRP was associated with a decrease in *Porphyromonas gingivalis* after six weeks [58], while reductions in this keystone pathogen were also observed at 24 h and three months following glycine-based interventions [57,58]. Erythritol-based protocols demonstrated an immediate decrease in *Porphyromonas gingivalis* post-treatment [21]. These transient reductions align with findings from earlier investigations [32,43]. In addition, *Tannerella forsythia* counts decreased after erythritol use at six months [56] and after glycine application at three months [57].

Host factors also appear to influence microbial outcomes. More favorable modifications in bacteria presumptively associated with periodontitis have been reported in female patients and non-smokers compared to males and smokers [57]. This finding is consistent with the immunosuppressive action of smoking [63] and gender-related differences in oral microbiota composition, as men generally present a higher prevalence of *Porphyromonas* and *Capnocytophaga* species [61].

The reductions in orange- and red-complex bacteria observed in some studies [21,56,57,59,61] were frequently temporary, with detection levels increasing to baseline within weeks. This may reflect methodological limitations, such as sampling intervals of 90 days, which could overlook short-term fluctuations, or the biological delay between recolonization and clinical impact. Furthermore, microbial persistence may involve complexes beyond the red complex, and relatively shallow pockets in some trials might have limited the extent of bacterial removal [57].

An immediate rise in bacterial counts following erythritol use has been reported, likely due to structural disintegration of the biofilm, including structures formed by *Aggregatibacter actinomycetemcomitans*, leading to transiently higher detected counts [21].

## 5. Effects of Air-Polishing Powders on Human Cell Response

Four in vitro studies that investigated the effect of different air-polishing powders on human oral cells were included in the present study (Figure 1).

### 5.1. Gingival Fibroblasts

In vitro studies have shown that air-polishing powders can negatively influence important functions of gingival fibroblasts, such as proliferation, viability, and wound healing capacity [15,31,42].

Recent work has evaluated the effects of glycine- and trehalose-based powders on fibroblasts from healthy donors. Glycine powder showed a pro-apoptotic effect, as seen by the increased expression of caspase-3 (an apoptotic marker) and lower levels of Proliferating Cell Nuclear Antigen (PCNA), which are indicative of reduced cell proliferation and wound healing. In contrast, trehalose powder had little effect on cell viability or proliferation [15].

Glycine also significantly increased some proinflammatory and angiogenic cytokines, such as Tumor Necrosis Factor alpha (TNF-α), Interleukin-8 (IL-8), C-C motif chemokine ligand 2 (CCL2), and Vascular Endothelial Growth Factor (VEGF). This suggests activation of the Nuclear Factor kappa-light-chain-enhancer of activated B cells (NF-κB) signaling pathway, which is known as a major driver of inflammation. On the other hand, trehalose did not cause significant cytokine changes, and thus it does not seem to activate the NF-κB pathway [15].

Another study compared glycine with an erythritol/chlorhexidine powder on fibroblasts obtained from three donors and evaluated cell viability after 12 and 24 h, proliferation at 24 and 48 h, and wound healing by scratch assay over 48 h. Erythritol/chlorhexidine notably reduced the number of live cells at 12 and 24 h and completely inhibited cell proliferation. Glycine also reduced gingival fibroblast proliferation, but to a lesser degree. These negative effects were linked to the chlorhexidine component, since pure erythritol caused the least impact on fibroblast viability compared to the control group [31]. Both glycine and erythritol/chlorhexidine powders significantly delayed wound closure compared to untreated controls [31].

Interestingly, erythritol showed anti-senescence properties, similar to N-acetylcysteine (a known senescence inhibitor). Erythritol inhibited the expression of senescence markers such as p16, p21, IL-1β, and TNF-α. This effect may be related to changes in cellular metabolism, including enhanced production of pyruvate, which is an important intermediate in the glycolytic pathway [64].

### 5.2. Dental Pulp Stem Cells

Human dental pulp stem cells cultured on titanium disks pretreated with glycine or tagatose maintained their ability toward osteogenic differentiation, confirmed by the expression of Runt-related transcription factor 2 (RUNX-2) and Osteocalcin (OCN) markers. VEGF expression was also increased after 7 days, suggesting a role in angiogenesis and bone formation [35]. These results are in agreement with previous studies reporting on the biocompatibility of glycine and tagatose on human dental pulp stem cells [42]. On the other hand, glycine significantly reduced the viability of gingival epithelial cells [42].

Trehalose was identified as a favorable powder to be used in air-polishing approaches, as it did not activate proinflammatory pathways or inhibit wound healing, showing a cell response profile compatible with optimal repair [15].

Oral and periodontal cells react differently to air-polishing powders. This variability highlights the importance of choosing the right powder to improve clinical outcomes and reduce tissue side effects [15,35,42]. Overall, the available in vitro evidence suggests that the biological effects of air-polishing powders on human oral cells are highly variable and dependent on both the composition of the powder and the cell type. Erythritol alone appears to exert fewer harmful effects and may even confer anti-senescence properties, but when used in conjunction with chlorhexidine, its cytotoxicity to fibroblasts is significantly increased [31,64]. Glycine, although widely used and compatible with dental pulp stem cell differentiation, has demonstrated proapoptotic and proinflammatory effects in gingival fibroblasts and epithelial cells, raising concerns about its potential to impair wound healing [15]. Trehalose has the most favorable profile, with minimal interference in cell viability, proliferation, or inflammatory signaling, suggesting low biological risk [15]. Tagatose also supports the osteogenic potential of stem cells and angiogenic responses, which could be beneficial for tissue repair [42]. These findings highlight the need for a balanced assessment; while all powders offer mechanical disruption of the biofilm, their different cytotoxic or proinflammatory effects are biological risks that need to be considered when choosing a type of air-polishing powder.

The predominantly positive microscopic effects of oral powders such as glycine and erythritol support their continued use in clinical practice, provided that precise protocols are followed to minimize potential complications. In contrast, for other powders such as tagatose and trehalose, further evidence and well-designed studies are required before they can be widely adopted in daily practice, especially in comparison with conventional powders.

## 6. Impact of Air-Polishing Powders on Local Tissues: Histological Observations from Experimental Models

These findings are supported by two ex vivo studies that investigated the influence of air-polishing on the oral gingiva in porcine models (Figure 1).

During air-polishing, some powder particles may impact soft tissues and cause localized damage due to the high pressure generated by the device [23,24,65].

In a porcine model, experimental teeth were polished using calcium carbonate particles (54 µm), sodium bicarbonate particles (65 µm), or erythritol particles (14 µm). Histological assessment of gingival excisional biopsies revealed that sodium bicarbonate and calcium carbonate caused epithelial alterations without penetrating the underlying connective tissue. These changes included degenerative epithelial modifications and mild desquamation of the superficial layer. Erythritol air-polishing resulted in only slight mucosal edema and minimal epithelial desquamation. Scanning electron microscopy (SEM) confirmed these findings, assigning a lesional score of 2 (disruption of superficial epithelial layers) to calcium carbonate and sodium bicarbonate, while erythritol received a score of 1, indicating minor superficial epithelial disruption [24].

An ex vivo study on porcine mandibles evaluated 25 µm glycine and 15 µm tagatose powders applied at either the manufacturer-recommended angle (30–60°) or 90° for 5 s at a 5 mm distance from the gingival tissue. Histological examination showed that most gingival samples treated with either powder at a 90° angle displayed no detectable damage. Conversely, at a 30–60° working angle, 25% of samples treated with tagatose exhibited connective tissue alterations, while 67% of those treated with glycine showed significant epithelial and/or connective tissue damage. SEM findings corroborated these histological observations [50].

Experimental evidence suggests that tissue impact during air-polishing depends on powder type, particle size, application angle, and pressure. Erythritol appears to cause the least epithelial disruption, while glycine and tagatose may induce more significant changes when applied at shallow angles [24,50]. Optimizing device settings and operator technique is essential to minimize soft-tissue injury [26,27].

## 7. Future Directions

The inconsistent results found regarding the effects of air-polishing powders on subgingival microbiota in periodontitis patients should be interpreted in the context of methodological differences and host-related biological factors. Most trials adopted comparable sampling protocols. However, variability exists across several dimensions: timing or intervention site (active therapy versus maintenance phase [57,59], pocket [21,56,60,61,62], or furcation sites [59]), subgingival strategies (air-polishing alone [57], before SRP [58], or after SRP [21,56,58,59,60,61]), supragingival applications [62], and follow-up schedules (immediately post-treatment [21,59,60,62]; at 24 h [58], 6 weeks [58], 3 months [56,61], or 6 months [56,57,59,60]). Some favorable microbiological changes have been documented. Sodium bicarbonate air-polishing resulted in an immediate reduction in total viable counts and in the prevalence of red and orange complex species [62]. Nevertheless, given the remaining uncertainties regarding the true antimicrobial benefits of air-polishing, further well-designed studies targeting these outcomes are still required, employing standardized protocols and accounting for host-related factors.

The interpretation of existing evidence is further complicated by methodological weaknesses within the included clinical studies. Many trials were limited by small sample sizes [21,59], restricted periodontal staging [21,61], short follow-up durations [58], or lack of appropriate control groups [56]. Some studies used non-standardized randomization methods, such as coin tosses in small samples [59], increasing potential bias. In addition, microbiological assessments were often restricted to a narrow range of pathogens [60], lacking comprehensive molecular approaches capable of capturing full microbial community shifts. The long-term stability of microbiological changes was seldom assessed, and several trials did not evaluate the influence of modifiable risk factors [60] or adjunctive therapies that may affect periodontal healing dynamics.

In vitro and ex vivo studies have provided important insights into the effects of air-polishing powders on soft tissues; their findings are limited by an absence of host factors such as vascularization, immune response and natural healing capacity [24,31].

Future systematic reviews and meta-analyses are needed to clarify these subjects. Moreover, future research should directly compare the effects of different air-polishing powders on oral cells, since cell-type-specific responses may vary in terms of cytotoxicity, oxidative stress, and cytokine release. Methodological aspects such as dose–response relationships, exposure time, particle size and hardness, as well as application pressure, should be carefully standardized to establish thresholds that minimize cellular damage while maintaining antibiofilm efficacy. Both clinical and in vitro studies are needed to clarify the above-mentioned aspects, using consistent protocols to assess clinical parameters, subgingival plaque samples, and microbiological outcomes. In addition, rigorously designed long-term clinical trials (lasting at least 6–12 months) are essential to confirm the safety and effectiveness of air-polishing powders in periodontal care. Standardizing the use of visual analog scales for assessing patient comfort would also be beneficial.

## 8. Conclusions

Air-polishing powders remain an effective option for the mechanical disruption of oral biofilm. Factors related to the physical properties of air-polishing jet streams, powder characteristics, and techniques of use could largely influence the efficacy of air-polishing approaches and the potential side effects on soft tissues.

Current evidence indicates that air-polishing powders may provide additional but variable and transient antimicrobial or anti-inflammatory effects. Glycine- and erythritol-based powders have been linked to reductions in *Porphyromonas gingivalis* at follow-up periods of up to three months in certain studies. Nevertheless, decreases in orange- and red-complex species after treatment often appear temporary, with recolonization to baseline levels occurring within weeks. Sodium bicarbonate is also an effective antibacterial agent, showing bactericidal activity against oral pathogens.

Laboratory studies indicate that air-polishing powders may impair gingival fibroblast functions such as proliferation, viability, and wound healing. Oral cell types do not respond uniformly to these powders, underlining the need for careful powder selection to induce optimal clinical and biological outcomes.

Overall, air-polishing appears to be a useful adjunct for rapid biofilm disruption during both active and supportive therapy.

### Limitations

This review has some limitations that should be acknowledged. The limited data identified based on our inclusion criteria preclude the statistical analyses necessary for a systematic review and meta-analysis. The literature search was restricted to only three databases and covered a relatively short timeframe, including only studies published in the last five years. This choice was intentional in order to capture the most recent evidence, but it may have led to the exclusion of relevant older studies. Moreover, some methodological inconsistencies were observed among some of the included studies, and given the relatively recent developments, expanding indications on air-polishing powders, the risk of publication bias should be considered. In addition, the evidence base is heterogeneous, with wide variation in study designs, powder formulations, and device settings, which limits comparability across studies and precludes robust meta-analysis.

## Figures and Tables

**Table 2:** Summary of microbiological findings from air-polishing studies.

Study (Year)	Powder Used	Study Type/Sample	Key Microbial Outcomes	Analysis Method
**Jentsch et al., 2020 [56]**	Erythritol + CHX	RCT, 42 periodontitis patients	Rare *A. actinomycetemcomitans*; *P. gingivalis* ↓ in control at 3 mo; *T. forsythia* & *T. denticola* ↓ at 6 mo in erythritol group; no intergroup difference	Multiplex real-time qPCR
**Sekino et al., 2020 [57]**	Glycine	RCT, 19 maintenance patients	↓ *P. gingivalis*, *T. forsythia* trends up to 90 days; *T. denticola* non-significant reduction; no intergroup differences	Real-time PCR with hybridization
**Zhang et al., 2020 [58]**	Glycine	RCT, 41 stage II-IV periodontitis patients	↓ *P. gingivalis* & *A. actinomycetemcomitans* at 24 h and 3 mo; *F. nucleatum* ↓ in glycine groups; *P. gingivalis* significantly reduced only when glycine before SRP at 6 wks	Real-time PCR
**Ulvik et al., 2021 [59]**	Erythritol + CHX	RCT, 20 maintenance patients with furcations	No intergroup differences; ↑ *T. denticola* & *P. micra* at 6 mo in both groups	Checkerboard DNA-DNA hybridization
**Wenzler et al. 2021** **[17]**	Glycine Trehalose	*In vitro*, Animal model	Total bacterial load ↓ 99% (glycine), ↓ 93.3% (trehalose), ↓ 77.6% (water); ↓ *P. gingivalis*, *F. nucleatum*, *C. rectus*, *A. actinomycetemcomitans*, *P. intermedia*	Real-time PCR
**Schulz et al., 2022 [60]**	Erythritol	RCT, 40 stage III-IV periodontitis patients	↓ *Fusobacterium* and uncultured *Prevotella* post-treatment, recolonization by 6 mo	16S rRNA sequencing, QIIME, alpha/beta diversity
**Mensi et al., 2022 [61]**	Erythritol + CHX	RCT, 40 stage III-IV periodontitis patients	↓ *T. denticola* at 3 mo in test; reductions in *A. israelii*, *F. alocis*, *P. endodontalis*, *T. forsythia*, *T. socranskii* mainly in females and non-smokers	qPCR microarray (93 species)
**Rams & Slots, 2023 [62]**	Sodium bicarbonate	CCT, 15 stage III periodontitis patients	84.9% ↓ total viable counts; ↓ *Prevotella intermedia*/*nigrescens*, *F. nucleatum*, red/orange complex species; motile morphotypes ↓ by 85.3%	Microbial culture, and phase-contrast microscopy
**Elmeligy et al., 2024 [21]**	Erythritol	RCT, 24 stage I-II periodontitis patients	Immediate ↑ *A. actinomycetemcomitans*; ↓ *P. gingivalis* post-treatment; no further changes	Culture, morphology and biochemistry

Abbreviations: *A*. *actinomycetemcomitans*, *Aggregatibacter actinomycetemcomitans*; *A. israelii*, *Actinomyces israelii*; *C. rectus*, *Campylobacter rectus*; CCT, Controlled clinical trial; CHX, clorhexdine; DNA, Deoxyribonucleic acid; *F. alocis*, *Filifactor alocis*; *F. nucleatum*, *Fusobacterium nucleatum*; h, hours; mo, months; PCR, polymerase chain reaction; *P. endodontalis*, *Porphyromonas endodontalis*; *P. gingivalis*, *Porphyromonas gingivalis*; *P. intermedia*, *Prevotella intermedia*; *P. micra*, *Parvimonas micra*; rRNA, Ribosomal ribonucleic acid; RCT, Randomized clinical trial; *T. forsythia*, *Tannerella forsythia*; *T. denticola*, *Treponema denticola*; *T. socranskii*, *Treponema socranski*; wks, weeks; ↑, Increase; ↓, Decrease.

## Data Availability

No new data were created or analyzed in this study. Data sharing is not applicable to this article.
